# Mesenchymal Stem Cells Alleviate Crohn's Disease by Regulating CBS‐Mediated Ferroptosis

**DOI:** 10.1002/iid3.70506

**Published:** 2026-09-21

**Authors:** Zhixi Huang, Xiaofang Xu, Zhou Huang, Bing Han, Dan Jiang, Xiaodan Lv, Ziqian Huang, Guangfu Lin, Fengxiang Huang, Yu Li, Lichun Han, Deyi Chen, Jianing Lin, Xiaoping Lv

**Affiliations:** ^1^ Department of Gastroenterology The First Affiliated Hospital of Guangxi Medical University Nanning China; ^2^ Department of Gastroenterology Guangxi Hospital Division of The First Affiliated Hospital of Sun Yat‐sen University Nanning China; ^3^ Department of Emergency The First Affiliated Hospital of Guangxi Medical University Nanning China; ^4^ Department of Clinical Experimental Medicine The First Affiliated Hospital of Guangxi Medical University Nanning China

**Keywords:** CBS, Crohn's disease, ferroptosis, mesenchymal stem cells

## Abstract

**Background:**

Mesenchymal stem cells (MSCs) possess immunoregulatory properties and have been shown to alleviate symptoms of Crohn's disease (CD). While ferroptosis has been implicated in the onset of CD, the precise connection between MSCs, CD, and ferroptosis remains unclear. This study sought to explore whether MSCs could alleviate CD by inhibiting ferroptosis.

**Materials and Methods:**

We screened the hub genes involved in both ferroptosis and CD and validated them in clinical colon biopsies by immunohistochemistry (IHC). MSCs were administered intraperitoneally to mice with colitis induced by trinitrobenzene sulfonic acid (TNBS) enema to evaluate the therapeutic effect. Additionally, a rescue experiment was conducted by co‐treating colitis mice with a cystathionine‐β‐synthase (CBS) inhibitor (aminooxyacetic acid, AOAA) and MSCs to further explore the mechanism of the MSCs‐CBS‐ferroptosis axis. The expression levels of CBS and ferroptosis‐related markers in colonic tissues were assessed by real‐time quantitative polymerase chain reaction (RT‐qPCR), western blot, and IHC.

**Results:**

Bioinformatics and machine learning identified CBS as a hub gene in both CD and ferroptosis. IHC revealed that CBS expression was downregulated in colon tissues from CD patients. MSCs treatment significantly reduced pathological symptoms and intestinal inflammation in colitis mice. MSCs increased CBS expression, which had been diminished in colitis mice. Additionally, MSCs increased glutathione (GSH) and glutathione peroxidase 4 (GPX4) expression, thereby inhibiting ferroptosis activation. In a rescue experiment, co‐treatment with AOAA and MSCs exacerbated intestinal inflammation and reversed the elevation of CBS and ferroptosis‐related markers.

**Discussion:**

This study demonstrates that MSCs alleviate the symptoms of CD by regulating CBS‐mediated ferroptosis.

## Graphical abstract

MSCs alleviate CD by regulating CBS‐mediated ferroptosis. Bioinformatics analysis identified CBS as a hub gene shared by CD and ferroptosis. In TNBS‐induced colitis mice, MSCs treatment alleviated intestinal inflammation by upregulating CBS expression, increasing GSH and GPX4 levels, and inhibiting ferroptosis. Conversely, co‐treatment with the CBS inhibitor AOAA reversed these protective effects.

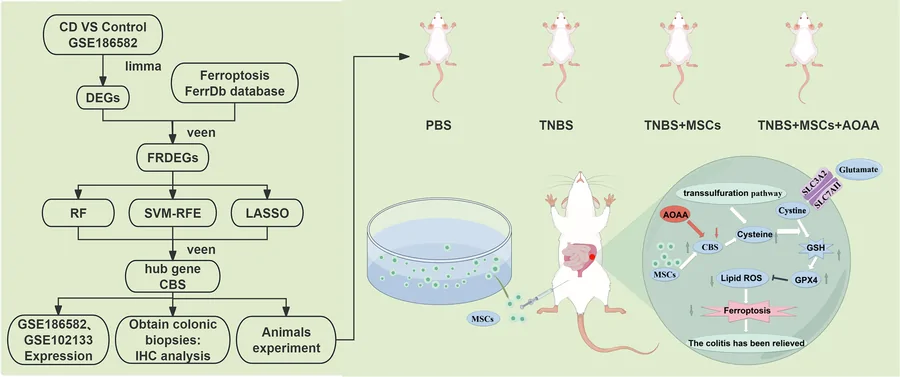

## 1 Introduction

Crohn's disease (CD) is a type of inflammatory bowel disease (IBD) that primarily affects the right colon and terminal ileum, causing symptoms such as diarrhea, abdominal pain, and intestinal obstruction [1]. While the exact cause of CD remains unclear, factors like immune system defects, microbial imbalances, and genetic predisposition are believed to contribute to its onset. Approximately 12% of CD patients have a genetic predisposition, with certain populations, including German Jews and Asians, being at higher risk [2]. Current treatments include corticosteroids, thiopurines, and biologics [3]. Fecal microbiota transplantation (FMT) has also been explored, but its effectiveness requires further validation [4]. Since no cure exists, understanding the disease's pathophysiology and exploring potential therapies is critical.

Mesenchymal stem cells (MSCs) are multipotent progenitor cells that can differentiate into various cell types [5]. MSCs can be isolated from sources such as amniotic fluid, umbilical cord, adipose tissue, bone marrow, and others. Their immunomodulatory properties are primarily driven by paracrine activity and direct cell‐immune cell contact [6]. MSCs regulate immune responses by releasing growth factors and cytokines, promoting angiogenesis, and preventing fibrosis [7]. They also suppress the proliferation and activation of key immune cells like macrophages, dendritic cells (DCs), natural killer (NK) cells, B cells, and T cells by arresting their cell cycles in the G0/G1 phase [8]. MSCs have shown promise in healing perianal fistulas in CD patients, but the exact mechanisms remain unknown [9].

Ferroptosis is a form of regulated cell death that depends on iron and is triggered by lipid peroxidation. This process occurs when the system xc^−^‐glutathione (GSH)‐glutathione peroxidase 4 (GPX4)‐dependent antioxidant system becomes inactive [10]. Cysteine, the rate‐limiting precursor of GSH synthesis, plays a key role in preventing lipid hydroperoxide accumulation [11]. The enzyme in the transsulfuration pathway that limits the rate is called cystathione‐β‐synthase (CBS). It has been shown that when cysteine is scarce, certain cells biosynthesize cysteine from methionine via the transsulfuration pathway [10]. CBS inhibitors can induce or enhance ferroptosis in various cell types [12, 13]. Aminooxyacetic acid (AOAA), a common inhibitor of CBS, has been used to block CBS activity both in vitro and in vivo [14, 15]. Ferroptosis has been linked to CD, as seen in elevated lipid hydroperoxides and impaired GPX4 activity in small intestinal epithelial cells (IECs) of CD patients. Inhibiting ferroptosis has also reduced disease severity in CD mouse models [16]. MSCs have been shown to prevent ferroptosis in the spinal cord [17] and liver injuries [18]. Our previous research demonstrated that MSCs can alleviate mice with colitis [19]. Therefore, we speculate that MSCs may alleviate CD intestinal inflammation by regulating CBS‐mediated ferroptosis.

In this study, we validated the beneficial effects of MSCs in CD mouse models, demonstrating how MSCs prevent ferroptosis and thereby reduce CD severity. These findings provide insights into the therapeutic potential of MSCs and contribute to a better understanding of their mechanisms in CD management.

## 2 Materials and Methods

### 2.1 Bioinformatics Analysis to Identify Hub Genes

The GEO database (https://www.ncbi.nlm.nih.gov/geo/) was searched using the term “Crohn's Disease” to identify relevant datasets. We retrieved the GSE186582 training dataset, annotated on the GPL570 platform, which includes 196 inflamed ileal samples from CD patients (M0I) and 25 ileal biopsy samples from healthy controls. Additionally, the GSE102133 validation dataset was downloaded and annotated on the GPL6244 platform, consisting of 12 intestinal mucosa samples from healthy individuals and 65 mucosa samples from CD patients. Ferroptosis‐related genes were obtained from the FerrDb database (http://www.zhounan.org/ferrdb), with the species parameter set to “Human”, yielding a total of 396 relevant genes.

Using the “limma” package in R, differentially expressed genes (DEGs) between the CD and control groups were identified from the training set. Genes with a *p* < 0.05 and |log2 fold change (FC)| > 1 were considered significant. Ferroptosis‐related genes were then intersected with the DEGs to identify ferroptosis‐related DEGs (FRDEGs) differentiating CD patients from healthy controls. FRDEGs were visualized using Venn diagrams, heatmaps, and volcano plots.

Hub genes within the FRDEGs were identified using three machine learning techniques. The least absolute shrinkage and selection operator (LASSO) algorithm was used to build a gene selection model and identify hub genes from FRDEGs, while the support vector machine recursive feature elimination (SVM‐RFE) algorithm selected hub genes by minimizing cross‐validation error. The importance of hub genes was also evaluated using the random forest (RF) algorithm. The top five genes from these methods were intersected, and the results were displayed in a Venn diagram. The GSE102133 dataset was used to validate hub gene expression. The diagnostic performance of the hub genes was assessed using the receiver operating characteristic (ROC) curve in both the training and validation sets.

### 2.2 Collection of Clinical Specimens

Pathological sections from CD patients were obtained from the pathology department of the First Affiliated Hospital of Guangxi Medical University. Diagnosis followed the 2018 Beijing Consensus on Inflammatory Bowel Disease, which includes clinical manifestations, laboratory tests, imaging, endoscopy, and pathology criteria [20]. Patients diagnosed with CD who had complete clinical follow‐up data were included in the study. Control intestinal tissue samples were also collected during the same period, with inclusion criteria being good health, absence of endoscopic inflammation, and no family history of IBD. Exclusion criteria included unclear diagnoses, other autoimmune diseases, recent intestinal diseases, or severe concurrent conditions. The clinical sample data are summarized in Table 1. Ethical approval for this study was obtained from the hospital's Ethics Committee (2024‐E431‐01).

**Table 1:** Summary of clinical sample information.

	Control	CD	*p*
*n*	25	25	
Age (mean ± SEM)	37.32 ± 2.286	31.44 ± 2.728	*p* = 0.1050
Sex	Male (*n* = 13)	Male (*n* = 13)	*p* > 0.05
	Female (*n* = 12)	Female (*n* = 12)	
Disease activity		Active period (*n* = 9)	
		Remission (*n* = 16)	
Disease severity		Mild (*n* = 3)	
		Moderate (*n* = 5)	
		Severe (*n* = 1)	
		Remission (*n* = 16)	
Lesion location		colon (*n* = 11)	
		Terminal ileum (*n* = 14)	
Complication		Anal fistula (*n* = 4)	
		Ileus (*n* = 2)	

### 2.3 Immunohistochemical (IHC) Analysis

Colon tissues from clinical samples and mice were fixed in 4% paraformaldehyde, embedded in paraffin, and sectioned into 4‐micrometer slices. After deparaffinization and hydration, antigen retrieval was performed using sodium citrate repair solution. Sections were incubated overnight at 4°C with primary antibodies anti‐CBS (1:50; Santa, USA) and anti‐GPX4 (1:200; Abmart, China). After incubating with an enzyme‐labeled anti‐mouse/rabbit IgG polymer (Zsbio, China), perform DAB staining (Zsbio, China), counterstain with hematoxylin (Zsbio, China), and bake the slides. Examine tissues with an intelligent pathology microscope system and capture images. The total positive staining area was calculated using ImageJ Fiji's batch macro.

### 2.4 MSCs Culturing and Animal Experiments

MSCs derived from BALB/c mice bone marrow (MUBMX‐01001) and a complete culture media kit (MUBMX‐90011) were purchased from Cyagen Biotechnologies (Guangzhou, China), which also supplied the MSCs identification report. Following the manufacturer's instructions, cells were cultured at 37°C in a humidified incubator with 5% CO_2_, using complete medium with 10% fetal bovine serum (Cyagen, China) and 1% penicillin‐streptomycin (NCMbio, China). When cell density reached 80–90% confluence, adherent cells were passaged.

Eight‐ to 9‐week‐old female BALB/c mice (19–21 g) were purchased from Guangdong Vital River and acclimatized for at least 1 week before experiments. Mice were housed under standard conditions with a 12‐h light/dark cycle and a controlled temperature of 20°C–22°C. Animal experiments were approved by the Guangxi Medical University Animal Care and Welfare Committee (Approval Number: 202209019).

A trinitrobenzene sulfonic acid (TNBS) (Sigma‐Aldrich, St. Louis, MO, USA)‐induced colitis mouse model was established following the method of Stefan‐Wirtz et al. [21]. Briefly, mice were pre‐sensitized with 150 μL of a 1% (w/v) TNBS‐acetone‐olive oil solution. Seven days later, they fasted for 24 h. The mice were then given an enema containing 2.5% (wt/vol) TNBS solution based on their body weight. Control mice received phosphate‐buffered saline (PBS) or an olive oil‐acetone solution enema. Mice were randomly assigned into four groups: control group, model group (TNBS + PBS), treatment group (TNBS + MSCs), and inhibitor group (TNBS + MSCs + AOAA). MSCs (1 × 10^6^/100 μL PBS) [19] were injected intraperitoneally on Days 1, 3, and 5 after model establishment as previously described [22]. The inhibitor group received AOAA (10 mg/kg/day) [23, 24] intraperitoneally for 5 days starting on Day 1, along with MSCs injections on Days 1, 3, and 5. Mice were weighed, and their disease activity was evaluated daily by assessing stool consistency, weight loss, and stool samples. A disease activity index (DAI) was calculated from these measurements [21] (Table 2). On day 8, mice were euthanized under isoflurane sedation, and colon tissues were collected and photographed. All live mice were included in the experiment and subsequent statistical analysis. The colon macroscopic damage index (CMDI) was assessed following established methods [25] (Table 3). The collected colon tissues were then used for further analysis. The experimental design is illustrated in Figure 3A.

**Table 2:** Disease activity index (DAI) scoring criteria.

Score	Weight loss	Stool consistency	Blood
0	None	Normal	Negative hemocult
1	1%–5%	Soft but still formed	Negative hemocult
2	6%–10%	Soft	Positive hemocult
3	11%–18%	Very soft; wet	Blood traces in stool visible
4	> 18%	Watery diarrhea	Gross rectal bleeding

**Table 3:** Colon mucosal damage index (CMDI) scoring criteria.

Score	Appearance
0	Normal
1	Localized hyperemia, no ulcers
2	Ulceration without hyperemia or bowel wall thickening
3	Ulceration with inflammation at one site
4	2 or more sites of ulceration and inflammation
5	Major sites of damage extending > 1 cm along length of colon
6–10	When an area of damage extended > 2 cm along length of colon, score was increased by 1 for each additional cm of involvement

We were blinded to group allocation during the conduct of the experiment, outcome assessment, and data analysis. Group assignments were coded by an independent researcher, and the codes were not revealed until all data collection and statistical analyses were complete.

### 2.5 Hematoxylin and Eosin (H&E) Staining

Mouse colon tissues were fixed in 4% paraformaldehyde, embedded in paraffin, and sectioned into 4‐micrometer slices. Sections were dehydrated in ethanol and stained with hematoxylin and eosin for light microscopy. Images were captured using an intelligent pathology microscopy system (Olympus Corporation, Japan). H&E staining was evaluated based on criteria established in the literature [26] (Table 4).

**Table 4:** HE scoring criteria.

Inflammatory cell infiltration		Tissue damage	
No infiltration	0	No mucosal damage	0
Increased number of inflammatory cells in the lamina propria	1	Discrete epithelial lesions	1
Inflammatory cells extending into the submucosa	2	Erosions or focal ulcerations	2
Transmural inflammatory cell infiltration	3	Severe mucosal damage with extensive ulceration extending into the bowel wall	3

### 2.6 Biochemical Detection

Levels of iron in colon tissue were assessed using an iron assay kit (Njjcbio, China), while reduced GSH and malondialdehyde (MDA) levels were measured using GSH and MDA kits (Gracebio, China). Tissue samples were homogenized in an ice bath, and the supernatant was collected for analysis. Absorbance was measured using a microplate reader (BioTek, USA), and concentrations of GSH, MDA, and iron in colon tissue were calculated according to the manufacturer's instructions.

### 2.7 Enzyme‐Linked Immunosorbent Assay (ELISA)

Levels of TNF‐α and IL‐10 in the mouse colon tissue homogenates were measured using ELISA kits for mouse TNF‐α and IL‐10 (DEVELOP, China), following the manufacturer's protocols.

### 2.8 Real‐Time Quantitative Polymerase Chain Reaction (RT‐qPCR) Analysis

Total RNA was extracted from tissues using RNAiso (Takara, Japan). The mRNA was reverse‐transcribed using a kit from Genstar (China). Relative gene expression levels in colon tissues were quantified using RT‐qPCR, with GAPDH as a control. Primer sequences are provided in Table 5.

**Table 5:** Primers used for RT‐qPCR analysis.

Primer name	species	Primer sequence
GPX4‐F	mouse	GCCTGGATAAGTACAGGGGTT
GPX4‐R	mouse	CATGCAGATCGACTAGCTGAG
XCT‐F	mouse	CCTCTGCCAGCTGTTATTGTT
XCT‐R	mouse	CCTGGCAAAACTGAGGAAAT
CBS‐F	mouse	CAACCCTTTGGCACACTACG
CBS‐R	mouse	TCCTTCAGCTTTCTGGCGAT
GAPDH‐F	mouse	AGGTTGTCTCCTGCGACTTCA
GAPDH‐R	mouse	GGGTGGTCCAGGGTTTCTTA

### 2.9 Western Blot

Tissue protein was extracted by adding protein lysate (RIPA with PMSF or protease inhibitor, 100:1) to the samples. Protein concentrations were determined using a BCA kit (Beyotime, China). Following high‐temperature denaturation, proteins were separated by SDS‐PAGE and transferred to a polyvinylidene fluoride (PVDF) membrane (Merck Millipore, Germany), which was then blocked with 5% skim milk. The membrane was incubated with the primary antibody overnight at 4°C. The membrane was then washed and incubated with secondary antibodies for 1 h at room temperature. Protein bands were visualized using the Odyssey two‐color infrared fluorescence imaging system (LI‐COR, USA). The primary antibodies used were anti‐β‐actin (1:1200; PTMab, China), anti‐FTH1 (1:1200; Abmart, China), anti‐GPX4 (1:1200; Abmart, China), anti‐CBS (1:700; Santa, USA), and anti‐SLC7A11 (1:1200; Abmart, China). Secondary antibodies included goat anti‐rabbit IgG (1:20000; Invitrogen, USA) and goat anti‐mouse IgG (1:20000; Invitrogen, USA).

### 2.10 Statistical Analysis

All data were analyzed using GraphPad Prism 9.5.1. Results are expressed as means ± SD. A *t*‐test was used to compare two groups, while one‐way ANOVA was employed for comparisons between multiple groups. Statistical significance was set at *p* < 0.05.

## 3 Results

### 3.1 CBS Is the Hub Gene for CD and Ferroptosis

**Figure 1:**
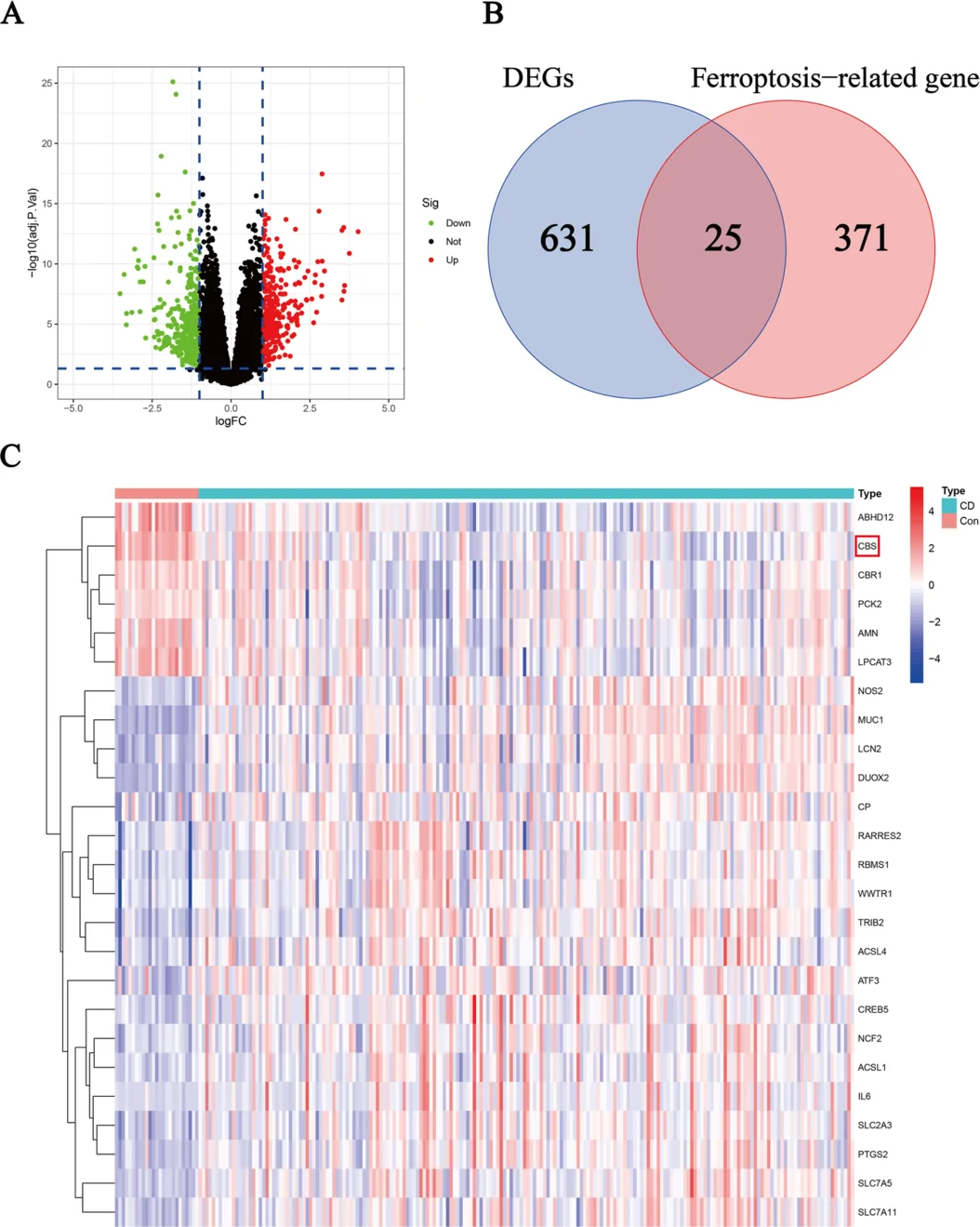
Identifying ferroptosis‐related DEGs (FRDEGs) in Crohn's disease (CD). (A) Volcano plot of differentially expressed genes (DEGs) generated from differential gene expression analysis of the GSE186582. (B) Venn diagram showing 25 FRDEGs obtained by intersecting DEGs with 396 ferroptosis‐associated genes. (C) Expression differences in FRDEGs between colonic tissues from CD patients and healthy individuals.

Gene expression analysis of the training set GSE186582 revealed 335 downregulated and 321 upregulated DEGs (Figure 1A). By intersecting these DEGs with 396 ferroptosis‐related genes, we identified 25 FRDEGs, 19 of which were upregulated and 6 downregulated (Figure 1B,C).

**Figure 2:**
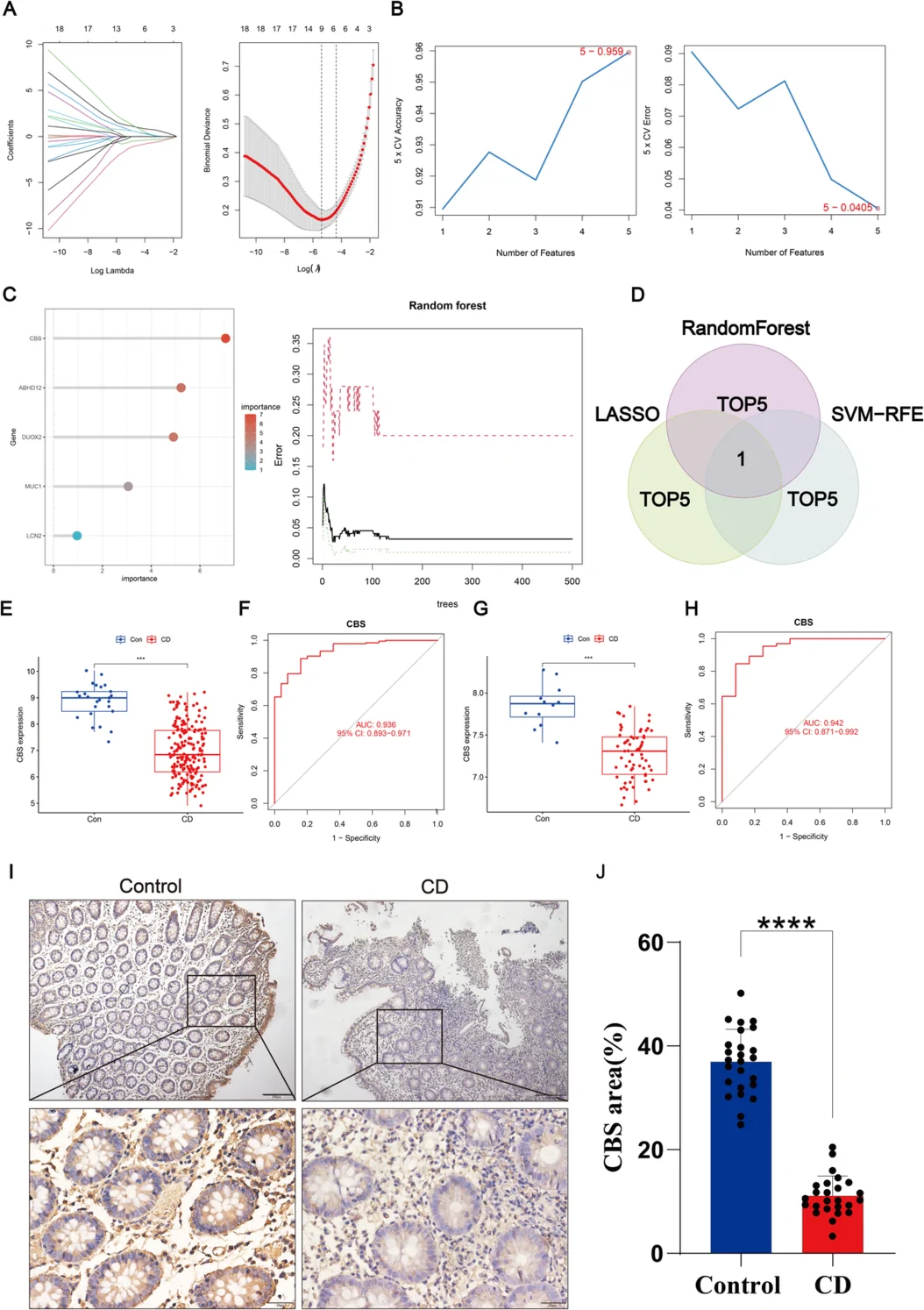
Screening hub genes from FRDEGs using machine learning. (A) The least absolute shrinkage and selection operator (LASSO) algorithm identified 9 genes from the FRDEGs. (B) The support vector machine recursive feature elimination (SVM‐RFE) algorithm identified 25 genes from the FRDEGs. (C) The random forest (RF) algorithm identified 25 genes from the FRDEGs. (D) The top five genes from each of the three machine learning methods intersected to identify cystathionine‐β‐synthase (CBS) as the hub gene. (E) Box plot of CBS expression in the GSE186582. (F) Receiver operating characteristic (ROC) curve for CBS in the GSE186582. (G) Box plot of CBS expression in the GSE102133. (H) ROC curve for CBS in the GSE102133. (I) Representative images of clinical samples stained for CBS using immunohistochemistry (IHC), with scale bars of 100 μm and 20 μm. (J) Quantification of CBS from IHC staining (n = 25). Mean ± SD are presented. A t‐test was applied. ^ns^ *p* > 0.05, *p < 0.05, **p < 0.01, ***p < 0.001, ****p < 0.0001.

Using three different machine learning algorithms, we identified potential hub genes from the FRDEGs. The LASSO algorithm identified 9 genes (Figure 2A), the SVM‐RFE algorithm identified 25 genes (Figure 2B), and the RF algorithm identified 25 genes (Figure 2C) (Table 6). CBS emerged as the hub gene, appearing among the top five genes from each of the three machine learning methods (Figure 2D). CBS, a ferroptosis‐inhibiting gene, was found to have downregulated expression in CD samples (Figure 2E). ROC curve analysis showed that CBS had an area under the curve (AUC) value of 0.936 (0.893–0.971) (Figure 2F). Validation using the GSE102133 dataset confirmed the downregulation of CBS in CD samples compared to healthy controls (Figure 2G). The AUC value for CBS in the validation set was 0.942 (0.871–0.992) (Figure 2H). Both the training and validation sets demonstrated consistent CBS expression levels, highlighting the gene's strong diagnostic potential.

**Table 6:** Genes identified by the LASSO, SVM‐RFE, and RF algorithms.

LASSO		SVM‐RFE		RF	
Gene	Coef	FeatureName	AvgRank	Gene	importance
(Intercept)	−13.165	NOS2	2.9	CBS	7.045453
MUC1	1.263875	CBS	3	ABHD12	5.223044
CBS	−1.56264	MUC1	5.4	DUOX2	4.91258
ABHD12	−0.73737	RARRES2	7.3	LPCAT3	4.618045
SLC7A11	−0.05423	SLC7A11	8	AMN	3.507098
NOS2	1.70982	LPCAT3	8.2	MUC1	3.062102
RARRES2	0.895489	LCN2	9.2	SLC7A5	2.357345
ACSL4	−0.32371	DUOX2	10.1	CREB5	2.316482
CBR1	0.076278	ACSL4	10.1	NOS2	1.702508
ATF3	0.700494	ATF3	10.9	ATF3	1.658639
		WWTR1	12.2	IL6	1.24427
		PCK2	12.6	PTGS2	1.021608
		NCF2	12.8	LCN2	0.96769
		AMN	12.9	NCF2	0.931548
		SLC2A3	14.3	PCK2	0.800853
		CREB5	15.9	RBMS1	0.793734
		IL6	15.9	CP	0.611187
		CP	17.5	SLC2A3	0.590353
		RBMS1	17.6	TRIB2	0.510752
		ACSL1	18.1	WWTR1	0.390435
		ABHD12	18.8	ACSL4	0.298538
		PTGS2	18.8	SLC7A11	0.295238
		SLC7A5	20.1	CBR1	0.253968
		TRIB2	20.6	RARRES2	0.238516
		CBR1	21.8	ACSL1	0.168376

### 3.2 CBS Expression Is Downregulated in the Colonic Tissue of CD Patients

IHC analysis showed that CBS is predominantly expressed in the cytoplasm (Figure 2I). Furthermore, CD patients exhibited significantly lower CBS expression levels compared to healthy individuals, consistent with the bioinformatics analysis (Figure 2J).

### 3.3 MSCs Attenuate Intestinal Inflammation and Pathological Symptoms in Colitis Mice

**Figure 3:**
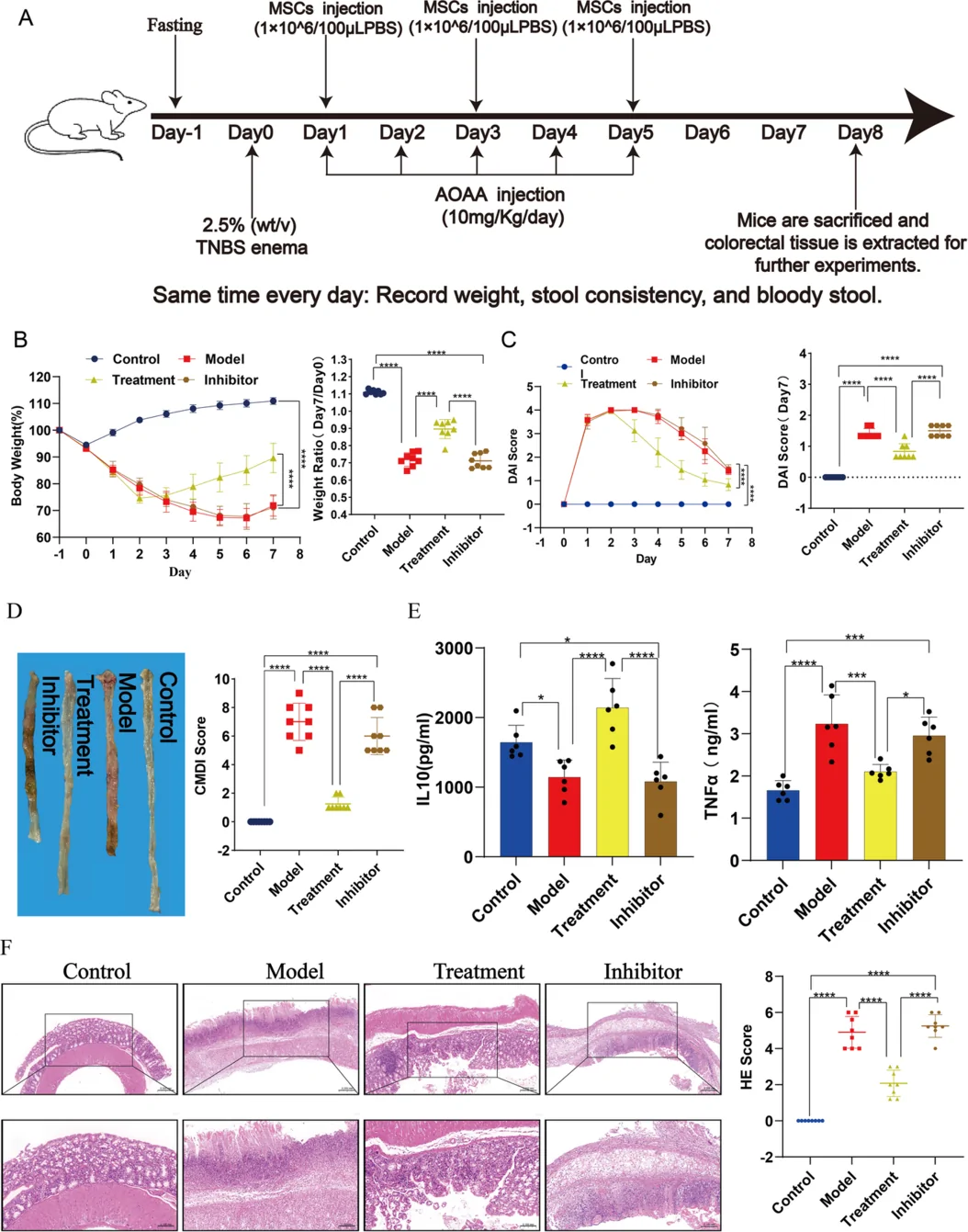
Mesenchymal stem cells (MSCs) attenuate intestinal inflammation and pathological symptoms in colitis mice. (A) Schematic diagram of trinitrobenzene sulfonic acid (TNBS)‐induced CD mouse models. (B) Daily body weight changes and comparison of weight on Day 7 versus Day 0 (*n* = 8). (C) Disease activity index (DAI) scores on Day 7 and daily fluctuations in DAI (*n* = 8). (D) Colon macroscopic observations and colon macroscopic damage index (CMDI) scores (*n* = 8). (E) Tissue levels of inflammatory cytokines (*n* = 6). (F) Histological inflammation scores and representative hematoxylin and eosin (H&E)‐stained images (*n* = 8); scale bars at 200 μM and 100 μM. Mean ± SD are presented. ANOVA was applied. ^ns^ *p* > 0.05, *p < 0.05, **p < 0.01, ***p < 0.001, ****p < 0.0001.

The TNBS‐induced colitis model is widely used as an animal model for CD [27, 28]. After receiving the TNBS enema, BALB/c mice experienced significant weight loss and increased DAI scores, confirming the successful induction of the colitis model. Mice treated with MSCs showed notable improvement in both weight loss and DAI scores. DAI scores naturally decreased over time due to the body's self‐repair mechanisms (Figure 3B,C). The model group showed more severe ulcers and higher CMDI scores, but these symptoms were alleviated following MSCs treatment (Figure 3D,F). MSCs treatment also increased the expression of the anti‐inflammatory factor IL‐10 and decreased the expression of the pro‐inflammatory factor TNF‐α compared to the model group (Figure 3E). Collectively, MSCs effectively reduced both the pathological symptoms and intestinal inflammation in the colitis mice.

### 3.4 MSCs Inhibit Ferroptosis in Colitis Mice

**Figure 4:**
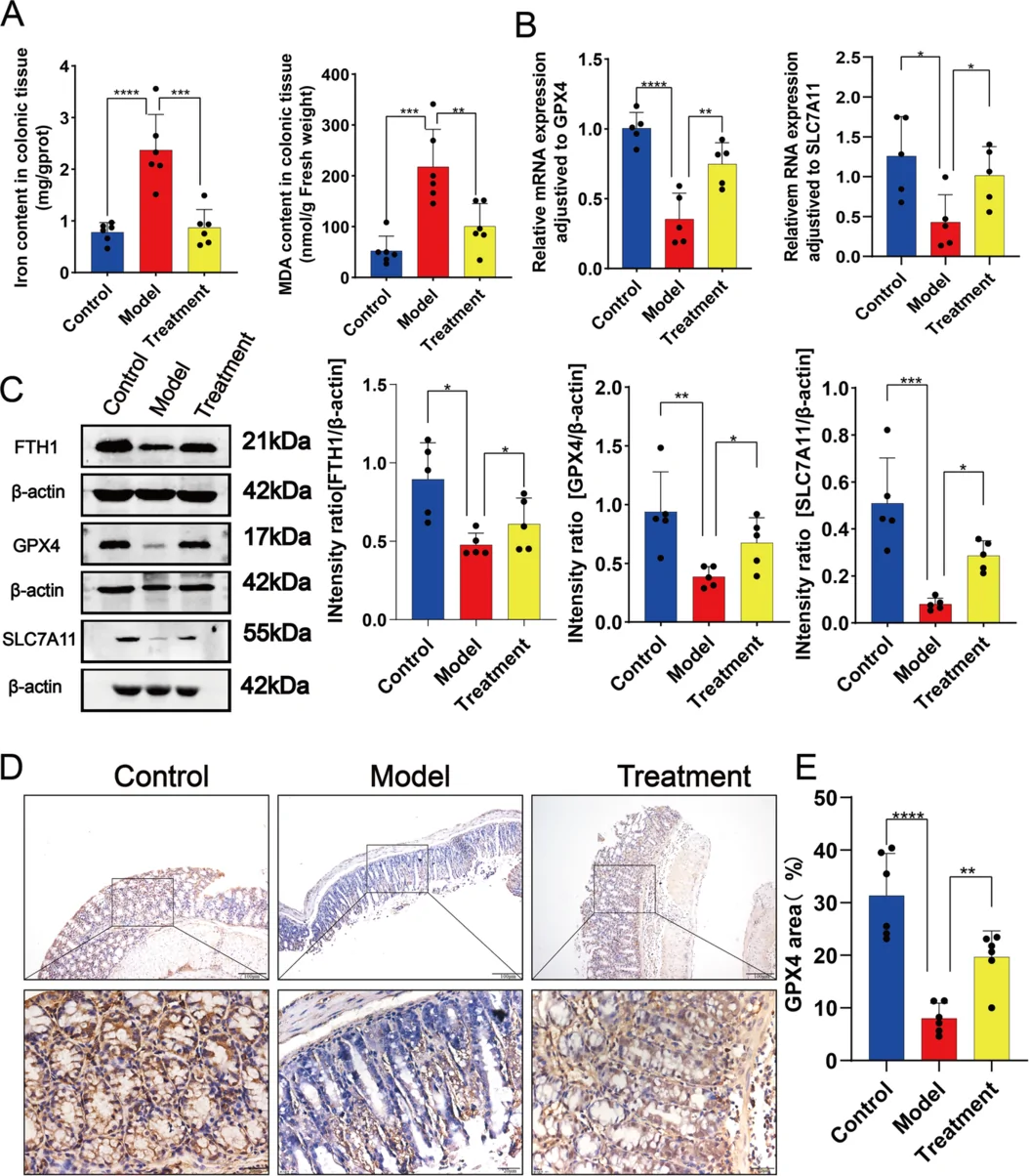
MSCs inhibit ferroptosis in colitis mice. (A) Levels of malondialdehyde (MDA) and iron content in colon tissues (*n* = 6). (B) Solute carrier family 7 member 11 (Slc7a11) and glutathione peroxidase 4 (GPX4) mRNA expression levels in mouse colon tissues were determined using real‐time quantitative polymerase chain reaction (RT‐qPCR) (*n* = 5). (C) Western blot analysis of ferritin heavy chain 1 (FTH1), GPX4, and SLC7A11 protein expression in colon tissues (*n* = 5). (D) Typical photos of colon tissue stained with GPX4 IHC, with scale bars of 100 μm and 20 μM. (E) Quantification of GPX4 from IHC staining (*n* = 6). Mean ± SD are presented. ANOVA was applied. ^ns^ *p* > 0.05, *p < 0.05, **p < 0.01, ***p < 0.001, ****p < 0.0001.

To determine whether MSCs treat CD by inhibiting ferroptosis, we examined ferroptosis‐related markers in mouse colon tissues. While the model group exhibited increased levels of MDA and iron in colon tissue, these markers decreased significantly after MSCs treatment (Figure 4A). *Gpx4* and solute carrier family 7 member 11 (*Slc7a11*) mRNA levels were significantly decreased in the model group but increased with MSCs treatment (Figure 4B). Western blot analysis confirmed increased protein levels of ferroptosis‐related genes ferritin heavy chain 1 (FTH1), GPX4, and SLC7A11 after MSCs treatment in colitis mice, consistent with mRNA expression (Figure 4C). IHC results showed that GPX4 was mainly localized in the cytoplasm, with a lower positive area ratio in the model group, which increased after MSCs treatment (Figure 4D,E). These results indicate that MSCs effectively suppress the activation of ferroptosis in colitis mice.

### 3.5 MSCs Upregulate CBS in Colitis Mice

**Figure 5:**
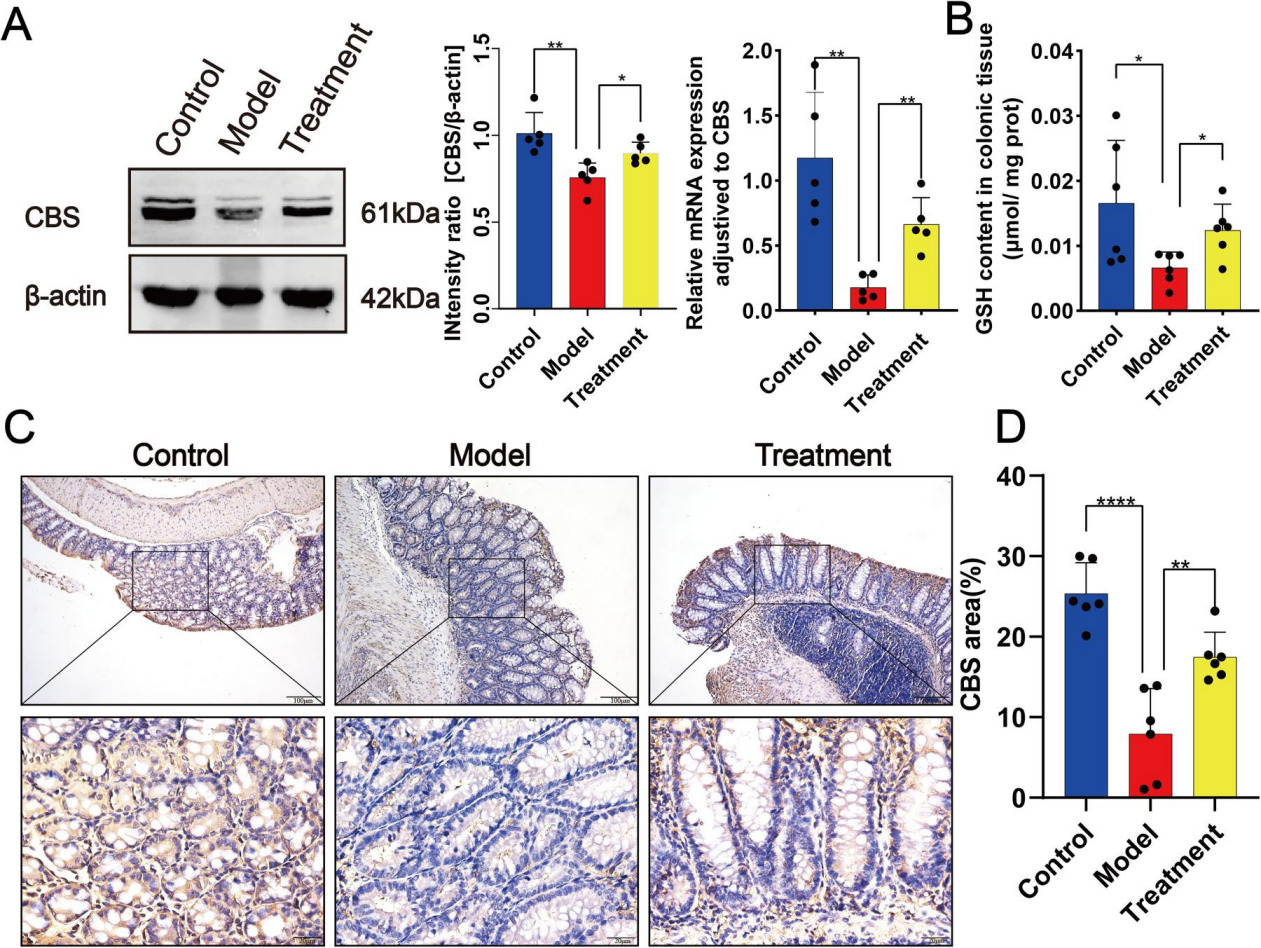
MSCs upregulate CBS in colitis mice. (A) Western blot and RT‐qPCR analyses of CBS mRNA and protein expression in colon tissue (*n* = 5). (B) GSH levels in colon tissue measured with a detection kit (*n* = 6). (C) Typical photos of colon tissue stained with CBS IHC, with scale bars of 100 and 20 μM. (D) Quantification of CBS from IHC staining (*n* = 6). Mean ± SD are presented. ANOVA was applied. ^ns^ *p* > 0.05, *p < 0.05, **p < 0.01, ***p < 0.001, ****p < 0.0001.

Recent studies suggest that CBS can counteract ferroptosis induced by oxidative stress [29]. In the absence of cysteine, CBS activates the transsulfuration pathway to generate cysteine [10]. To investigate whether MSCs prevent ferroptosis by upregulating CBS, we assessed the expression of CBS and GSH levels in mouse colon tissues. In comparison to the model group, the MSCs treatment resulted in significantly higher levels of CBS mRNA and protein expression (Figure 5A) as well as GSH levels (Figure 5B). IHC results showed that CBS was mainly localized in the cytoplasm, with a lower positive area ratio in the model group, which increased after MSCs treatment (Figure 5C,D). These findings suggest that MSCs upregulate CBS expression, thereby inhibiting ferroptosis.

### 3.6 MSCs Alleviate Colitis by Inhibiting CBS‐Mediated Ferroptosis

**Figure 6:**
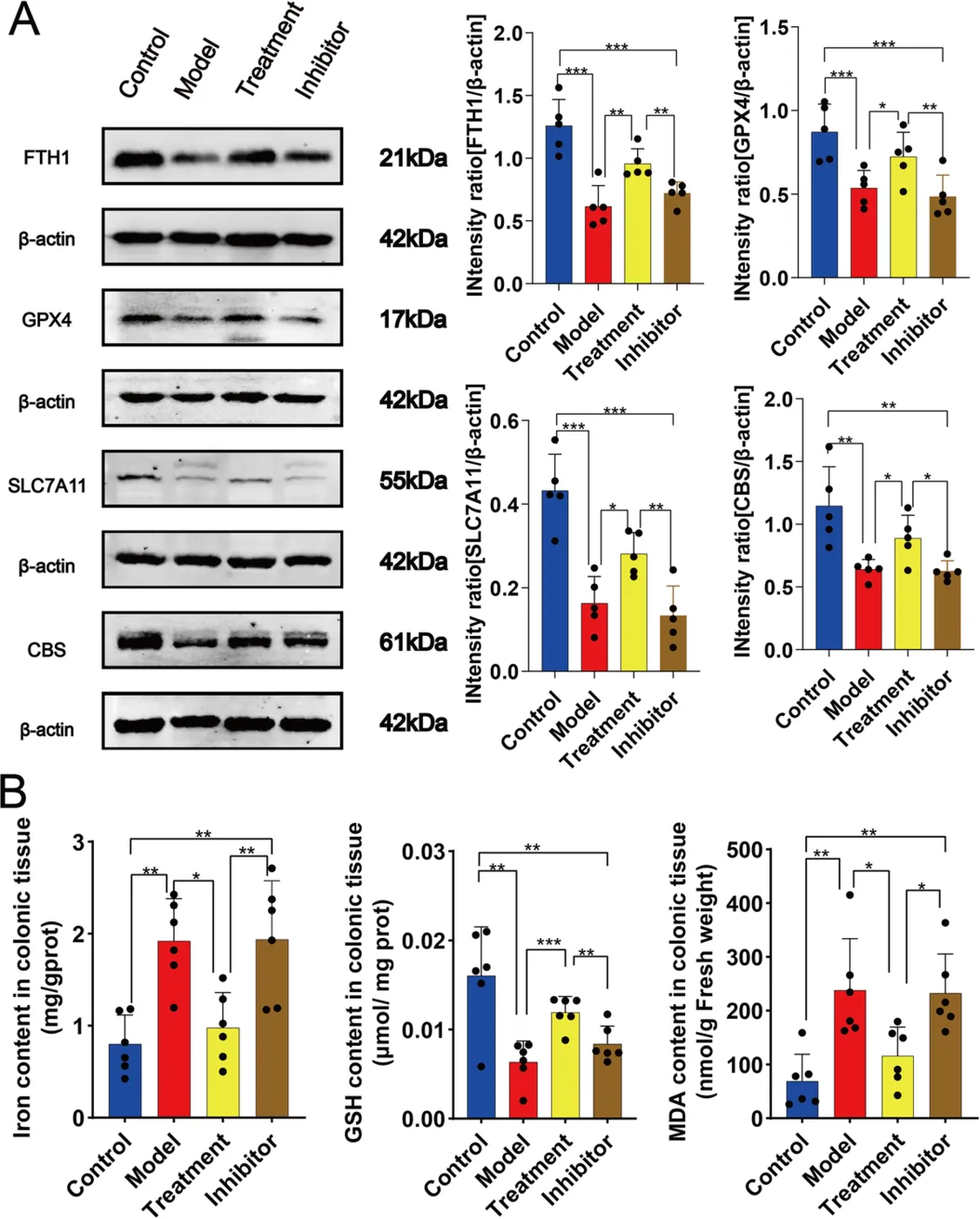
MSCs alleviate colitis by inhibiting CBS‐mediated ferroptosis. (A) Western blot analysis of CBS, GPX4, SLC7A11, and FTH1 protein expression in colon tissues (*n* = 5). (B) GSH, MDA, and iron content in colon tissue, measured with detection kits (*n* = 6). Mean ± SD are presented. ANOVA was applied. ^ns^ *p* > 0.05, *p < 0.05, **p < 0.01, ***p < 0.001, ****p < 0.0001.

To further explore the mechanism of the MSCs‐CBS‐ferroptosis axis, a rescue experiment was conducted by co‐treating colitis mice with a CBS inhibitor (AOAA) and MSCs. The inhibitor group experienced greater weight loss (Figure 3B) and higher DAI scores (Figure 3C) than the treatment group. In contrast to the treatment group, the inhibitor group exhibited higher TNF‐α expression levels and lower IL‐10 expression levels (Figure 3E). Moreover, the inhibitor group displayed more severe ulceration and elevated CMDI scores (Figure 3D,F). Protein expression levels of CBS, SLC7A11, FTH1, and GPX4 were lower in the inhibitor group than in the treatment group (Figure 6A). Additionally, GSH levels were significantly lower in the inhibitor group, while MDA and iron levels in tissue were higher (Figure 6B). These results indicate that MSCs alleviate colitis by inhibiting CBS‐mediated ferroptosis.

## 4 Discussion

In this study, we identified CBS as a hub gene in CD and ferroptosis through bioinformatics analysis and confirmed that CBS expression was downregulated in the colonic tissues of CD patients compared to healthy individuals. Animal experiments revealed that MSCs improved weight loss, pathological changes, and intestinal inflammation in TNBS‐induced colitis mice. Concurrently, MSCs increased the expression of CBS and suppressed the activation of ferroptosis in colitis mice. However, co‐treatment with a CBS inhibitor and MSCs reversed the aforementioned effects of MSCs.

Ferroptosis, a recently identified form of programmed cell death, has been implicated in the pathogenesis of various diseases, including cancer [30], liver injury [31], traumatic brain injury [32], and myocardial infarction [33]. The mechanisms regulating ferroptosis fall into three broad categories: iron metabolism, GPX4 regulation, and lipid metabolism. Most research to date has focused on iron overload and lipid peroxidation [34]. Our findings indicated that in colitis mice, reduced expression of SLC7A11, GSH, and GPX4 led to the inactivation of the system xc^−^‐GSH‐GPX4‐dependent antioxidant defense, increasing susceptibility to lipid peroxidation. Iron overload has long been associated with conditions such as spinal cord injury, cardiomyopathy, and alcoholic liver disease [35, 36, 37]. Moreover, excessive iron intake may exacerbate intestinal inflammation by intensifying the inflammatory response or altering the epigenetic profile of IECs [38, 39]. In our study, elevated iron levels and decreased FTH1 expression indicated iron overload in CD mouse models. Thus, ferroptosis in this model was triggered by a combination of iron overload and antioxidant inactivation. MSCs are known to migrate to damaged tissues and organs, releasing growth factors, cytokines, and exosomes to support regeneration [40, 41]. Ample evidence supports MSCs' role in inhibiting ferroptosis. For example, MSCs and their exosomes have been shown to mitigate acute spinal cord injury in mice by promoting ferroptosis inhibitor (FSP1) expression [42]. Human umbilical cord MSCs (HucMSCs) improve erectile dysfunction in diabetic rats by reducing ferroptosis [43]. Additionally, MSCs inhibit ferroptosis in myocardial ischemia‐reperfusion injury by upregulating GPX4, enhancing intracellular glutathione synthesis [44]. Since ferroptosis plays a crucial role in the onset and progression of CD, targeting this process could offer new therapeutic avenues for IBD [45]. Our study demonstrated that MSCs inhibit ferroptosis and alleviate CD in a mouse model. This is supported by the restoration of the antioxidant system and inhibition of ferroptosis, evidenced by increased expression of ferroptosis marker genes. After MSCs treatment, CD symptoms were alleviated, the DAI score decreased, and survival rates improved in the mouse models. Thus, we concluded that MSCs alleviate CD by inhibiting ferroptosis.

CBS plays a crucial role in cysteine production via the transsulfuration pathway, particularly when intracellular cysteine is limited or the system xc^−^ function is compromised, as is the case in ferroptosis. Our results showed that CBS expression is reduced in CD mouse models but increased following MSCs treatment. In the inhibitor group treated with both AOAA and MSCs, CBS expression was lower than in the treatment group, with no significant difference from the model group. Therefore, MSCs can upregulate CBS, stimulating the transsulfuration pathway to produce cysteine, which is essential for GSH synthesis. By increasing GSH and GPX4 expression, MSCs help reactivate the antioxidant system, thereby inhibiting ferroptosis. Concurrently, decreased CBS expression was observed in the colonic tissues of CD patients, suggesting that MSCs can alleviate CD by regulating CBS‐mediated ferroptosis.

It is important to recognize the limitations of this study. First, although the TNBS‐induced colitis model is widely used to mimic CD‐like intestinal inflammation, it does not fully replicate the chronic relapsing nature or immunological heterogeneity of human CD. Second, the mechanistic relied primarily on pharmacological inhibition of CBS with AOAA. Genetic approaches such as CBS knockdown or knockout in colonic tissues would provide more definitive evidence of the causal role of CBS in MSCs‐mediated ferroptosis inhibition. Third, while a range of ferroptosis‐related markers were assessed, direct ultrastructural evidence of ferroptosis, such as mitochondrial shrinkage and cristae loss via transmission electron microscopy, was not included. This would have provided further substantiation for the occurrence of ferroptosis.

Future research should address the limitations identified in this study and extend the findings in several directions. Translational studies using clinical‐grade MSCs in larger animal models or organoid systems must evaluate the efficacy, safety, and optimal delivery routes for CD treatment. From a mechanistic perspective, genetic manipulation of CBS in both MSCs and intestinal tissues will be essential to confirm the causal role of the CBS‐ferroptosis axis. Furthermore, integrating single‐cell RNA sequencing with spatial transcriptomics could help to elucidate the intricate dynamics of cellular communication between MSCs and colonic epithelial or immune cells in the context of ferroptosis regulation.

## 5 Conclusion

In conclusion, this study demonstrates that MSCs upregulate CBS expression, stimulate cysteine synthesis, restore the antioxidant system, inhibit ferroptosis, reduce inflammation, and thereby alleviate CD. These findings provide a new theoretical framework for understanding the pathogenesis and treatment of CD. Targeting ferroptosis inhibition through MSCs could represent a novel therapeutic approach for CD.

## Author Contributions

**Zhixi Huang:** conceptualization, methodology, investigation, validation, software, data curation, formal analysis, project administration, writing – review and editing, writing – original draft, visualization, supervision. **Xiaofang Xu:** writing – original draft, conceptualization, data curation, formal analysis, investigation, methodology, software, supervision, validation, visualization, writing – review and editing. **Zhou Huang:** data curation, formal analysis, writing – review and editing, conceptualization. **Bing Han:** writing – original draft, data curation, formal analysis, writing – review and editing. **Dan Jiang:** conceptualization, formal analysis, investigation, writing – review and editing. **Xiaodan Lv:** conceptualization, investigation, methodology, writing – review and editing. **Ziqian Huang:** investigation, methodology, project administration, writing – review and editing. **Guangfu Lin:** methodology, project administration, writing – review and editing, software. **Fengxiang Huang:** project administration, software, writing – review and editing. **Yu Li:** project administration, writing – review and editing, software, supervision. **Lichun Han:** validation, supervision, visualization, writing – review and editing. **Deyi Chen:** formal analysis, validation, writing – review and editing, visualization. **Jianing Lin:** data curation, software, supervision, writing – review and editing. **Xiaoping Lv:** writing – original draft, conceptualization, formal analysis, funding acquisition, investigation, methodology, project administration, resources, software, supervision, writing – review and editing.

## Ethics Statement

The study involving human participants was conducted in accordance with the principles outlined in the Declaration of Helsinki. Ethical approval was granted by the Ethics Committee of the First Affiliated Hospital of Guangxi Medical University (approval number: 2024‐E431‐01). Prior to participation, all individuals provided written informed consent after being thoroughly informed about the study's objectives, procedures, potential risks, and benefits, as well as their right to withdraw at any point without prejudice. Additionally, the animal experiments received approval from the Animal Care and Welfare Committee of Guangxi Medical University (approval number: 202209019).

## Conflicts of Interest

The authors declare no conflicts of interest.

## Data Availability

The data and materials used in this study are available from the corresponding author upon reasonable request.
